# Concepts of Suffering at the End of Life Amongst Emergency, Palliative Care and Geriatric Medicine Physicians in Malaysia

**DOI:** 10.1177/10499091251317725

**Published:** 2025-01-29

**Authors:** Annushkha Sinnathamby, Yun Ting Ong, Shu Xian Lim, Aaron Wi Han Hiew, Sing Yee Ng, Joyce Huimin Chee, Mark Kiak Min Tan, Nur Amira Binte Abdul Hamid, Simon Yew Kuang Ong, Lalit Kumar Radha Krishna

**Affiliations:** 1 Khoo Teck Puat National University Children’s Medical Institute, 150744 National University Health System, Singapore; 2 Division of Cancer Education, 68751 National Cancer Centre Singapore, Singapore; 3 Division of Supportive and Palliative Care, 68751 National Cancer Centre Singapore, Singapore; 4 Division of Supportive and Palliative Care, National University Cancer Institute Singapore, Singapore; 5 Palliative Care Unit, General Medicine Department, 58983 Hospital Kuala Lumpur, Kuala Lumpur, Malaysia; 6 Internal Medicine Department, 277090 Hospital Sultan Ismail, Johor Bahru, Malaysia; 7 Saw Swee Hock School of Public Health, 37580 National University of Singapore, Singapore; 8 Medical Ethics & Law Department, Faculty of Medicine, 54703 Universiti Teknologi MARA Sungai Buloh Campus, Selangor, Malaysia; 9 Division of Medical Oncology, 68751 National Cancer Centre Singapore, Singapore; 10 Duke-NUS Medical School, 37580 National University of Singapore, Singapore; 11 Health Data Science, 4591 University of Liverpool, Liverpool, UK; 12 Yong Loo Lin School of Medicine, 37580 National University of Singapore, Singapore; 13 Centre for Biomedical Ethics, 37580 National University of Singapore, Singapore; 14 Palliative Care Institute Liverpool, Academic Palliative & End of Life Care Centre, Cancer Research Centre, 4591 University of Liverpool, Liverpool, UK; 15 PalC, The Palliative Care Centre for Excellence in Research and Education, Singapore

**Keywords:** suffering, death and dying, palliative care, geriatrics, accident and emergency, costs of caring, burnout

## Abstract

**Background:**

Palliative Care, Geriatrics and Emergency physicians are exposed to death, terminally ill patients and distress of patients and their families. As physicians bear witness to patients’ suffering, they are vulnerable to the costs of caring—the emotional distress associated with providing compassionate and empathetic care to patients. If left unattended, this may culminate in burnout and compromise professional identity. This study aims to provide a better understanding of suffering across various practice settings and specialties to guide the design of support frameworks for physicians and their patients.

**Methods:**

From August 2023 to September 2024, semi-structured interviews were conducted with sixteen Palliative Care, 12 Geriatrics and 13 Emergency physicians from various hospitals in Malaysia. Interview transcripts were analyzed using both inductive and deductive qualitative analyses.

**Results:**

Data analysis revealed three key domains: (1) living and dying well, (2) definition of suffering, and (3) impact of patient suffering on physicians.

**Conclusion:**

Physicians’ concepts of a good life and death frame their notions of suffering beyond the antithesis of a good life. Suffering is found to be distress at a loss of control, independence and dignity, alongside the presence of physical, emotional and existential distress. Witnessing patient suffering predisposes to physician suffering as they question their goals and roles in patient care. Our findings underscore the need for host organizations, hospitals and clinical departments to invest more in the care of their physicians. We believe these findings ought to be applicable to many resource-limited nations and other health care professionals beyond Malaysian shores.

## Introduction

Palliative Care (PC), Geriatric Medicine (geriatrics) and Accident and Emergency (A&E) expose physicians to terminally ill patients, death and distress of patients and their families.^1-6^ Recent reviews on caring for terminally ill patients and their families have found that such exposure to patient and family suffering has a significant impact on the well-being and professional identity formation (PIF) of physicians^3-5,7-10^ — how they think, feel and act as professionals. With direct and indirect impacts on patient safety, family care, interprofessional working and physician welfare,^2,4,11-13^ the need to appreciate the effects of exposure to patients’ and their families’ suffering, delineated as *“states of severe distress associated with events that threaten the intactness of person”*,^11^ is clear.

Whilst how physicians contend with suffering is highly personalized, there is little by way of appreciating how different forms of suffering impact physicians. Superficially, we evaluated the possible differences caused by sudden death and shocked families among A&E physicians, anticipated and possibly slow dying processes on geriatricians and the demise of suffering patients among PC physicians.

## Methods

**Figure 1.**
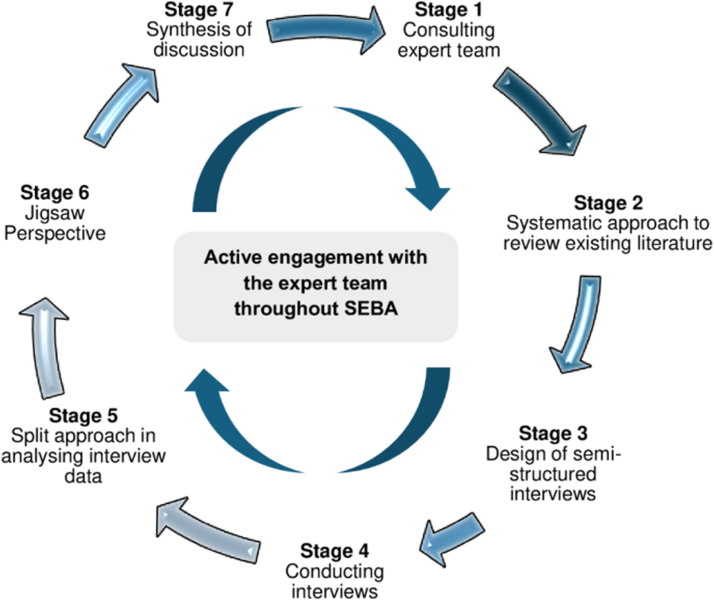
The Systematic Evidence-Based Approach (SEBA) Process, applied to primary studies.

To address our primary research question, *“What is known about the impact of suffering on Malaysian physicians?”*, and secondary question, *“What is known about the impact of suffering on the professional identities of Malaysian physicians?”*, the Systematic Evidence-Based Approach^3,14-19^ (SEBA) was adopted as the foundational framework that guided our research process. SEBA’s six-stage process for conducting qualitative interviews is shown in Figure 1.

### Ethics Approval and Consent to Participate

Ethical approval (NMRR ID: NMRR ID-23-00005-YY8 and Protocol ID: RSCH ID-22-01209-TSZ) was obtained from the Medical Research and Ethics Committee (MREC) of the Ministry of Health (MOH) Malaysia. Written and oral consent was obtained from all participants.

### Stage 1. Expert Team

The SEBA methodology recommends the use of an expert team to oversee the research process and provide a balanced review.^3,14^ This involved clinicians, clinician-educators and a medical librarian from an oncology centre, a palliative care institute and local medical schools collaborating jointly with a research team of research assistants and medical students.

#### Reflexivity

The research team was guided by palliative care consultants with expertise in medical education, qualitative analysis and systematic reviews that accorded them ‘insider’ roles privy to nuances, thus enhancing the rigor of the study. In safeguarding personal and group reflexivity in data interpretation, the research team consulted expert team members throughout the process to attenuate the impact of personal experiences and biases on the study. Further, to minimize social desirability bias, two trained interviewers who did not share working relationships with participants were recruited to conduct qualitative interviews.

The interviewer has 8 years of research experience with the University of Malaysia, and holds a Bachelor’s in Psychology and a Master’s in Management Psychology. She did not have conflict of interest with the participants. Her background assisted her in conduct of the interviews by enabling her to establish rapport with participants and maintain a structured approach, ensuring consistency and reliability in data collection.

### Stage 2. Systematic Approach

In crafting a semi-structured interview guide, the expert and research teams undertook a systematic approach to review extant literature on the care of terminally ill patients in Malaysia.^20,21^

### Stage 3. Design of Semi-Structured Interviews

Data from the reviews informed the design of the interview guide, which sought to capture how the personal beliefs, values and practices of physicians were impacted by their lived experiences. The interview guide was subsequently reviewed and revised by local palliative care and oncology physicians and qualitative researchers using a modified Delphi process.^22^ The interview guide is enclosed in Online Additional File 1 below.

### Stage 4. Conducting Semi-Structured Interviews

Purposive sampling guided the dissemination of email invitations that detailed the aim and nature of the study to physicians in local hospitals in Malaysia. Voluntary and anonymous participation was emphasized and participants were free to withdraw from the study at any point. Between 17^th^ August 2023 and 12^th^ September 2024, 16 physicians from PC, 12 physicians from geriatrics and 13 physicians from A&E were recruited for one-on-one interviews conducted by two trained research team members. Verbal and written consent were attained from all participants at the start of the interviews. Conducted in English on an institutionally secured Zoom video conferencing platform, each interview was audio-recorded and ranged from 30 minutes to an hour.

The research teams conducted data collection and analysis concurrently. Recruitment ceased when two consecutive interviews failed to yield any new insights. Each audio recording was transcribed verbatim, anonymized and sent to each participant for member checking to confirm that the transcripts accurately reflected their perceptions.^3^

### Stage 5. Split Approach

To facilitate a review from multiple viewpoints, two independent teams of at least two authors concurrently performed Hsieh and Shannon’s^23^ thematic analysis and Braun and Clarke’s^24^ directed content analysis, which drew from Kuek et al.’s^5^ and Ho et al.’s^12^ respective studies on caring for dying patients in intensive care units and physician-patient boundaries in palliative care. This simultaneous analysis further helped to offset the limitations of each method. Contradictory data and the omission of negative results in thematic analysis, for example, can be accounted for by content analysis.

In analyzing the data independently, each team member constructed codes from the interview transcripts and uncovered key subthemes from the derived codes. Larger themes were then created from the identified sub-themes. The teams exchanged broader patterns of meaning and maintained audit trails.^3^ The team members practiced Sandelowski and Barroso’s^25^ approach to *“negotiated consensual validation”* to achieve consensus on the key themes and categories.

### Stage 6. Jigsaw Perspective

Guided by Phases Four to Six of France et al.’s^26^ adaptation of Noblit and Hare’s^27^ seven phases of meta-ethnography, the Jigsaw Perspective saw the merging of similar or overlapping categories and themes to form key domains. Reciprocal translation was employed to determine whether the themes and categories could be used interchangeably.

#### Iterative Process

A review of the results by the expert and research teams revealed the delineation of new findings. This was highlighted in the proffering of Domain 3, which was related to physicians’ suffering.

## Results

Table 1 summarizes the demographic details of the physicians who participated in the study.

**Table 1.** Participant Demographics.

Palliative Care Participants	Years of Work	Geriatric Care Participants	Years of Work	A&E Participants	Years of Work
P1	4	G1	19	E1	12
P2	4	G2	11	E2	10
P3	8	G3	14	E3	7
P4	3.5	G4	4	E4	15
P5	4	G5	10	E5	13
P6	11	G6	2	E6	15
P7	2.5	G7	2	E7	24
P8	10	G8	3	E8	N/A
P9	4	G9	11	E9	15
P10	5	G10	7	E10	12
P11	7	G11	6	E11	16
P12	5	G12	15	E12	10
P13	10			E13	10
P14	14				
P15	12				
P16	12				

### Domain 1. Living and Dying Well

Suffering is antithetical to living and dying well. Understanding the concepts of living and dying well helps platform understanding of suffering.

#### Living Well

Physicians described living well in seven dimensions:

1. Happiness and satisfaction.
2. Good quality of life, including good health and environment.
3. Independence and living life by one’s own values, beliefs, experiences and relationships.
4. Maintenance of dignity.
5. Living closely to one’s faith and spirituality and making appropriate preparation for the afterlife.
6. A meaningful life in the service of family and society, alongside making a difference to the community.
7. Achievement of personal goals and financial stability.

Representative quotes are as follows (full supporting quotes can be found in Online Additional File 2).

“I think the good life is seeking the pleasure of work, which of course involves this aspect of taking your share of this world, enjoying things that are enjoyable and pleasant in this world.” (P11)

“Good life means, for me… surrounded by family members, maintain a good relationship with friends and family.” (G4)

#### Dying Well

Dying well is similarly multi-dimensional and highly personalised to the patient and family’s beliefs.

“What a good dying should be like… people should reconcile with things before they leave this world. And there will be an intentional effort to try to explore their regrets or unfinished business.” (P7)

“If you could benefit the people around you, you could make your mark and leave a good impact to the world.” (E10)

Supporting quotes can be found in Online Additional File 3.

### Domain 2. Definition of Suffering

Like a good life and death, suffering is personalized and multi-faceted (P11, E1, E11, E12, G2, G8, G12). This includes the following concepts:

#### Emotional Suffering

Negative emotions include unhappiness (P4, P7, E8, E10, G2, G6, G11), a lack of hope (P4, P11) and helplessness (P4, P7, E8, E10, G2, G6, G11). It also includes mental health conditions, such as depression in the face of terminal illness (P2-P10, P12, P15, G1-G7, G9, G10, E2, E3, E7, E9, E10, E12).

#### Spiritual Suffering

Suffering spiritually or existentially (P11, P8, P10, P9, P7, P6, P5, P4, P3, P15, E12) comes into conflict with finding meaning in life (P11, P16), which may result in deep-seated frustration (P7).

#### Loss in Independence and Control

For some patients, a loss in independence (P12-P14, G2-G4, G6, G11, G12) and a lack of control over circumstances and treatments (P1-P3, P8, P12, P15, P16, G8, E5) is deemed as suffering.

They have lost something that they used to have and now, they are in a new state whereby they feel helpless. They cannot control things anymore and they are not aware or don’t know how long this will continue. The uncertainty of this. (P1)

For some patients, this then results as a loss in dignity (P12-P14, G2, G3, G4, G6, G11, G12):

The loneliness and the dignity. We say that because of the fact that you become dependent. The patient becomes dependent on others for their living. So, I think dignity, the loss of dignity is suffering. (G4)

Suffering from the loss of dignity is also precipitated by the lack of personalized support from physicians and patients’ families (P10, P9, P5, P4, P3, G2, G3, G6, E10, E11, E13) leading to a

Poor quality of life (P12-P14, G2-G4, G6, G11, G12, E13).

#### Social Suffering

This refers to the inability to interact with others or fulfill one’s social function (P2-P14, G1, G4, G5, G9, E2-E4), loneliness (P12-P14, G2-G4, G6, G11, G12) and lack of familial support (P2-P14, G1, G4, G5, G9, E2-E4). This can also be caused by deep-seated familial conflicts (G2, G6, G8, G12).

### Suffering in Terms of Physical Pain

Physicians across all three specialties recognized poor symptom control as a source of suffering (P2-P16, G1-G10, E1-E3, E7, E9, E10). This is especially so amongst A&E physicians, in part due to a lack of exposure to emotional suffering (E1, E2, E4-E9, E11-13):

Psychosocial? Let’s see. To my recollection, I have not seen a severe one though. I honestly have not seen a severe one. (E6)

### Domain 3. Impact of Patient Suffering on Physicians

SEBA’s iterative process proffered the notion that physician suffering is a result of patient suffering.

#### Precipitating Factors for Physician Suffering

Physician suffering arose from various sources.

##### Helplessness

The inability to help, save and/or cure patients while witnessing their suffering was one of the root causes of physician suffering:

When somebody’s suffering is more related to physical issues, it’s usually easier for us, medical practitioners, to respond in a way that makes sense quite easily… But when it comes to psychosocial, as well as spiritual suffering, we are sort of limited in a way that the answer to this kind of suffering is usually less related to medications or treatments… And the ability to relate to psychosocial and spiritual suffering as well is a bit more challenging because [I] may find it difficult to try and put myself in a position where I can try and understand what they're going through. (P10)

##### Ill-Equipped to Address the Complexity of Suffering

Some A&E physicians reported that they were ill-equipped, inadequately resourced, and ill-suited to contend with psycho-emotional suffering (E1), given the nature of the A&E setting. In addition, E2, E4, E9 and E12 recognized the need for input from psychiatry or palliative support:

You need team management, probably the counsellor or the psychiatrist, and to have some other people sit in. (E9)

##### Failure to Fully Consider Patient Suffering

Geriatricians (G4, G5, G10, P14) noted that psychological and/or existential suffering was, at times, given short shrift, much to the physicians’ regret:

I think this [psychological distress and suffering] is something that sometimes we actually, voluntarily or involuntarily, will try to brush aside. We won’t entertain much… But sadly speaking, these are the things that also will actually impact patients a lot. (G4)

Physician suffering was also compounded by failure to recognize the complex intertwined nature of suffering (P3, P7, P9, G2, E9).

#### Costs of Caring

The impact of patient suffering and their own suffering could leave physicians with long-lasting psycho-emotional distress (P3, P2, G2):

When you try to be in their shoes and you feel the same for them,… these emotions and thoughts will linger with you for a long time. (G2)

These ‘costs of caring’ might be exacerbated by transference, countertransference, blurred professional-patient boundaries (P2-P5, P14, P15, G7, E10) and meaningless suffering (P2, P11):

We don’t see the meaning and it’s quite easy not to see the meaning in our patients who are suffering at that point in time. But maybe because we’re just too distracted and not putting enough thought into it, not putting enough effort. (P2)

Such costs of caring might also be the product of a failure to acknowledge personal limitations (P1, P13, E11):

It is not our job to take away 100% [of the symptoms] ... It’s something I had to learn over the years. (P9)

The costs of caring also included impulsive and incorrect clinical decisions (P2) that might compromise care and/or difficulty in attending to patient suffering (P10, E9, E1):

I suppose it’s a bit more difficult, physical pain you have morphine and things like that. And psychological pain, I suppose, takes a bit of time to sit and talk and explain and answer. (E9)

Some physicians suffered a crisis in confidence (P3, E1, E12) and questioned their ability to attenuate suffering (P1, P13) and their role in clinical practice (P1-P3, G1, G5):

What am I doing? Am I actually helping the patient, or I’m just pushing the patient to an early grave? (E1)

Sometimes, I question myself, actually what have I done to help this person? … We are just there to witness, guide and probably accompany them along their journey. I don’t think we are able to change completely their experience, to be honest. (P1)

For some physicians, the costs of caring manifested as a lack of meaning in their work:

Finding meaning in your work, knowing that whatever happens at work, it is never personal. And to recognize that it is the patients and the family members who are suffering and that is why they are behaving the way they are. (P13)

Other physicians came to a sense of resignation and ‘acceptance’ as to the inevitability of suffering:

I have come to realise over the years, that this kind of suffering is something that a lot of people cannot do much about… A lot of things cannot be changed and a lot of things cannot be resolved. (G5)

## Discussion

This study provides several insights into the impact of patient suffering on physicians. To begin, the notions of a good life and death help frame current concepts of suffering and move the understanding of suffering beyond merely the antithesis of a good life. A good life considers independence and autonomous functioning, meaningful existence, dignity and freedom from pain and noxious stimuli. A good death continues along this thread, focusing on the dying process whilst the legacy of what has been earned, learned and lived by continues to impact those that follow. Acceptance of fate, putting affairs in order and preparing the family for one’s end are key to legacy building and dying on one’s own terms. Inferences to autonomy, control and dignity are apparent, couched in the desire to be free of distress and in the bosom of kith and kin.^28,29^ A patient’s suffering is then seen as unhappiness at a loss of control, independence and dignity, coupled with the presence of physical, emotional and existential distress.

Yet, the data also uncovers a critical form of suffering—physician suffering. Born out of a failure to recognize, address and ameliorate symptoms and re-establish function and independence, physician suffering is characterized by the physician’s goals and narratives behind their role in care. Helplessness despite having the intention to support, cure and care, along with being ill-equipped to achieve treatment goals, also uncover the ‘costs of caring’ extending beyond compassion fatigue, to include vicarious trauma and secondary traumatic stress.^30^ This is in concordance with other studies exploring the impact of death and dying on health care professionals.^30-45^ This distress is exacerbated by transference, countertransference, blurred professional-patient boundaries and witnessing meaningless suffering.^12^ Failure to meet clinical, personal, professional and the patient’s end-of-life goals cements this distress and compounds physician suffering.

Perhaps tellingly, these effects are cumulative, snowballing towards maladaptive practices if left unattended. Boundary setting and distancing from patients lead to a lack of empathy in care interactions, concealed within the hustle and bustle of A&E departments. The effects of suffering amongst PC physicians lead to blurring of patient-physician boundaries and eventually, to less-than-holistic and compassionate care.^30,46^ Burnout and attrition from the service are rising possibilities, as are growing mental health issues and concerns over well-being.^43-45,47-51^

As one physician puts it, *‘to care is to suffer, to survive is to see meaning in the suffering of those we care for and acceptance for those left behind’*. If ‘*caring is to suffer with those we journey with’* holds true, this study underscores the need for greater personalized, longitudinal and timely support of physicians in order for these critical services to continue attending to the neediest of patients and their families.^52,53^ Our findings underscore the need for host organizations, hospitals and clinical departments to invest more in the care of their physicians.^6,54-56^ We believe that while these findings draw on Malaysian physicians, their lessons ought to be applicable to many resource-limited nations and other health care professionals beyond Malaysian shores.

### Limitations

This study was conducted in a single country. To improve transferability, physicians from multiple specialties with various practice settings were interviewed. Further studies are required in different countries with unique sociocultural contexts to better compare and contrast physicians’ experiences in caring for dying patients. Additionally, the use of one-time interviews as the primary source of data in this study captured only the views of physicians at a single time point and restricted the depth and scope of being able to triangulate collected data when compared to multimodal methods of data collection. Further, longitudinal studies are required. Retrospective accounts are also susceptible to recall bias.

## Conclusion

As earlier studies have shown, the need for longitudinal and personalized assessments is key to timely and appropriate support of physicians if burnout and attrition amongst the ranks are to be attenuated and patient care, experiences, outcomes and safety are to be maintained. We hope to focus our attention on delving into longitudinal assessment methods to address these gaps as we look forward to continuing discussion in this critical aspect of effective care provision.

## Supplemental Material

Supplemental Material - Concepts of Suffering at the End of Life Amongst Emergency, Palliative Care and Geriatric Medicine Physicians in Malaysia Supplemental Material for Concepts of Suffering at the End of Life Amongst Emergency, Palliative Care and Geriatric Medicine Physicians in Malaysia by Annushkha Sinnathamby, Yun Ting Ong, Shu Xian Lim, Aaron Wi Han Hiew, Sing Yee Ng, Joyce Huimin Chee, Mark Kiak Min Tan, Nur Amira Binte Abdul Hamid, Simon Yew Kuang Ong, and Lalit Kumar Radha Krishna in American Journal of Hospice and Palliative Medicine®.

## Appendix

### Abbreviations

PC: Palliative Care
A&E: Accident and Emergency
PIF: Professional Identity Formation
SEBA: Systematic Evidence-Based Approach
MREC: Medical Research and Ethics Committee
MOH: Ministry of Health
