# Supplementary material for: Concepts of Suffering at the End of Life Amongst Emergency, Palliative Care and Geriatric Medicine Physicians in Malaysia
Source: Am J Hosp Palliat Care. 2025 Jan 29;43(1):85–93. doi: 10.1177/10499091251317725 (PMC12627250; doi:10.1177/10499091251317725)
Supplement: Supplemental Material - Concepts of Suffering at the End of Life Amongst Emergency, Palliative Care and Geriatric Medicine Physicians in Malaysia [file sj-pdf-1-ajh-10.1177_10499091251317725.pdf]

Additional File 1. Interview Guide

**Introduction**	• Hi __, thank you so much for taking the time to attend this interview.<br>• This will be a three-part study. We hope to understand your views on patient suffering, and death and dying, and subsequently, your views on physician-assisted suicide and euthanasia in Malaysia.<br>• In doing so, we seek to understand how these impacts your personhood, or 'what makes you, you' so that the institution can better support healthcare professionals like yourself.<br>• This interview will be audio recorded and anonymised for analysis. The audio recording will be deleted after the study is complete. During the interview, do try to refrain from stating names but in the event that you do, we will anonymise them as well. Do I have your permission to record and begin the interview?
**Topic Areas**	**Questions**
**Demographic Questions**	*To start off this interview, I would like to understand more about your background in medicine.*<br><br>1. Could you share what your speciality is in medicine?<br>2. Were there any particular personal values or beliefs that attracted you to medicine/ [this field]? → *highlight said values/beliefs again after the sharing of memorable cases to prompt if these beliefs were reinforced or challenged (to show synchrony/dyssynchrony etc.)*<br>3. How long have you been in your field?<br>4. Could you describe your typical day at work?
**Attitudes towards witnessing the suffering of patients/ Memorable case of suffering**	*Thank you for sharing. I understand that you have encountered countless number of patients in your line of work/in the span of your career. I imagine that having to witness their suffering is inevitable.*<br><br>1. What constitutes patient suffering to you?<br>2. What is the commonly observed nature of their suffering? *e.g. physical, psychosocial, spiritual etc.*<br>3. Do the different natures of their suffering impact you differently? i.e. do some forms of suffering carry more weight than others? Why?<br>4. Does seeing patients suffer affect you?<br>5. How does it affect you? *e.g. spiritual, emotional, psychosocial*<br>6. How does it impact your thinking and views about yourself as a healthcare professional?<br>7. How do you deal with these feelings?<br>8. What are the skills you possess that you believe are important in caring for suffering/dying patients?<br>9. Could you share with us a memorable case of suffering that has rocked your confidence, beliefs, or faith/ something that has left a lasting impact on you?<br>10. What were the patient's needs?<br>11. What were the loved ones' needs?

	12. How did you respond to their needs?<br>*13.* What were the most challenging aspects of this encounter? *e.g. spiritual, emotional, psychosocial*<br>14. How does caring for suffering/dying patients affect the way you think about your patient's family?<br>15. How did this affect your relationships with the people who matter to you?<br>• Some doctors / nurses we interviewed have said that caring for dying patients makes them distance themselves from their loved ones for fear of loss<br><br>16. Why was this case memorable to you?<br>17. How has this case impacted you? How have you adapted following this case?<br>18. Was there some conflict with what you believed in versus what was expected of you?<br>• For e.g., some doctors we had interviewed struggled with caring for suffering patients as they were questioning whether they were prolonging the dying processes rather than prolonging a good quality of life for the patient.<br><br>19. Have you experienced instances of moral distress in your line of work?<br>20. i.e., when you encounter situations where your personal moral/ethical values were in conflict with the needs, preferences or decisions of the patient, their family, the medical team, or the larger healthcare system?<br><br>*(If not already raised, go to questions under the innate / individual / relational / societal rings)*<br><br>**Possible prompts:**<br><br>21. **Innate Ring**: Did being a witness to a patient's suffering affect your perspective on a good life and death? / How did it affect your religious/spiritual beliefs (or vice versa)/ personal values?<br><br>22. **Individual Ring:** How did this case affect your perspective on palliative care/your day-to-day emotions/the way you see yourself/what you value in life?<br><br>23. **Relational Ring:** How did this affect your relationships with the people who matter to you?<br>• Some doctors / nurses we interviewed have said that caring for dying patients makes them distance themselves from their loved ones for fear of loss.<br>• Has this always been the case or has this changed in the course of caring for suffering/dying patients? (If it has changed, why?)<br><br>24. **Societal Ring**: How did this case affect your role as a physician? Did you wish or feel like you could have done more for the patient during their suffering?

	If the concept of **'dyssynchrony'** is brought up in discussion of the other rings → Go to **'Conflicts & Dyssynchrony'**.
**Identity Work/ Coping Strategies**	5. How have you adapted or changed following the case?<br>6. Did it affect your decision-making or your actions?<br>• For e.g., has this encounter led to compassion fatigue, helplessness, anger, influenced your thoughts about your career, or has the thought of quitting because the conflict was too great to bear crossed your mind?<br>• For e.g., have you had to give up your **r**eligion / something deeply important to you because of this encounter?<br>7. How did you deal with this? i.e., What helped you find an answer and balance? (Who or what helped you in achieving resolution?)<br>8. Has it changed along the way in the course of your career?<br>9. Do you find yourself seeking support from your colleagues/mentors/senior doctors? / *More general: What was your source of support during difficult patient encounters of suffering?*
**Impact of Death and Dying/ Memorable Case**	*Thank you for sharing, I understand that it's not always easy having to bear witness to a patient suffering and in many cases, it leads to the death of the patient.*<br><br>10. When you see and care for these dying patients, how does it impact your thinking about yourself as a healthcare professional?<br>• For some doctors, they feel a sense of helplessness when their patients pass on while for others, they feel a sense of relief knowing that their patients no longer have to suffer.<br>11. What aspect of seeing and caring for dying patients affects you the most?<br>12. How does this change when the patient dies?<br>13. How does their death impact you?<br>14. How do you deal with these feelings?<br>15. Does the death of a patient who had been suffering impact you differently?<br>• Is there a difference in how you feel and cope when a patient who has been suffering passes on vs the death of a patient who hasn't endured this period of suffering?<br>16. Could you share with us a memorable case of death and dying, maybe a case that either rocked or reinforced your confidence, beliefs or faith?<br>17. Why was this case memorable to you?<br>18. How have you adapted or changed following this case?<br><br>*(If not already raised, go to questions under identity work/coping strategies and innate / individual / relational / societal rings)*<br><br>**Possible prompts:**

	• **Innate Ring**: Did this case affect your perspective on a good life and death? / How did it affect your religious/spiritual beliefs (or vice versa)/ personal values?<br><br>• **Individual Ring:** How did this case affect your perspective on palliative care/your day-to-day emotions/the way you see yourself/what you value in life?<br><br>• **Relational Ring:** How did this affect your relationships with the people who matter to you?<br><br>• **Societal Ring**: How did this case affect your role as a physician?<br><br>If the concept of **'dyssynchrony'** is brought up in discussion of the other rings → go to 'Conflicts & Dyssynchrony'.

**Attitudes towards Euthanasia/ Memorable Case**	*Thank you for sharing that. Having discussed earlier about patients suffering and dying at the end of life, *would euthanasia or PAS ever present as a viable option to you as a healthcare professional?*<br><br>19. ***If yes,** what are your thoughts on euthanasia/PAS being illegal in Malaysia?<br>20. *If no, what about suffering/dying patients who have no other options available to them?<br>21. What are your thoughts/beliefs on: if it's legalized, what are your views?<br>• Euthanasia?<br>• PAS?<br>• Palliative Sedation?<br>**What are the driving factors behind their beliefs on these procedures? Religion? Sanctity of life? Freedom of choice? Culture? Medical advancements etc.?*<br><br>22. In what circumstance, if any, is euthanasia/PAS an acceptable option to you? What about non-treatment decisions?<br><br>23. What do you think are some common reasons behind a patient's request to undergo euthanasia?<br><br>*24.* How would you respond to a patient's request to undergo euthanasia? / What would you advise a patient who wants to proceed with euthanasia? → *did it challenge or reinforce their aforementioned beliefs on the issue?*<br><br>25. Have you ever witnessed a patient being euthanised or provided with PAS?<br>• Can you share more on this case?<br>• What was the main reason behind their request for euthanasia/PAS?<br>• What were their needs and their families' needs?<br>• How did it impact you?<br>• How did you deal with the effects and/or challenges? *(go to identity work/coping strategies)*<br><br>26. Does the death of a patient from natural causes impact you differently from a patient who has been euthanized? If so, how and why?<br><br>27. What was your opinion on euthanasia prior to the COVID Pandemic crisis?<br><br>28. Has it changed since then? If yes, how?<br><br>*(If not already raised, go to questions under the innate / individual / relational / societal rings)*<br>**Possible prompts:**<br><br>29. **Innate Ring:** How does euthanasia affect your religious/spiritual beliefs (or vice versa)?

30. **Individual Ring:** How did this case affect your perspective on palliative care/your day-to-day emotions/what you value in life?

31. **Relational Ring:**
    a. How would you feel if your loved one requested euthanasia/ assisted suicide? Why would you feel this way?
    b. If you were in the same situation as the patient, what would you like your loved ones to do for you?

32. **Societal Ring**: How does the existence of euthanasia affect your perspective on being a physician? What do you think about your colleagues/other physicians who are proponents of euthanasia and PAS?/ What do you think of the commonly purported view that physicians who advocate for euthanasia are trying to 'play God'?

Guide for interviewer

- ***Euthanasia*** is defined as follows: a physician (or other person) intentionally killing a person by the administration of drugs, at that person's voluntary and competent request.
- ***Physician assisted suicide (PAS)*** is defined as follows: a physician intentionally helping a person to terminate his or her life by providing drugs for self-administration, at that person's voluntary and competent request.
- ***Palliative* sedation** is defined as follows: a physician employs the use of sedative medication to reduce consciousness in dying patients (prognosis <2 weeks) and thereby relieve otherwise unbearable suffering from refractory symptoms
- ***Non-treatment decisions (NTD)*** is defined as follows: withholding or withdrawing medical treatment from a person either because of medical futility or at that person's voluntary and competent request.

<table>
<tr><td>Conflict & Dyssynchrony in Suffering, Euthanasia and Death & Dying</td><td>33. In the cases you have highlighted, was there some conflict with what you believed in versus what was expected of you?
For e.g., some doctors we had interviewed struggled with caring for suffering patients as they were questioning whether they were prolonging the dying processes rather than prolonging a good quality of life for the patient.
For e.g., because of your personal beliefs, do you agree with the use of supportive measures that may potentially prolong the dying process rather than improve the quality of life? Or even may potentially shorten life?
34. (Possible Follow-up) →
How do you feel about this conflict?
35. Have you experienced instances of moral distress in your line of work?
I.e., when you encounter situations where your personal moral/ethical values were in conflict with the needs, preferences or decisions of the patient, their family, the medical team, or the larger healthcare system?
Possible prompts:
Have you witnessed healthcare providers giving “false hope” to a patient or family?
Have you participated in care that caused unnecessary suffering or compromised the dignity of the patient?
Have you witnessed a violation of a standard of practice or a code of ethics and not felt sufficiently supported to report the violation?
Have you witnessed a member of your team being disrespectful or abusive to a patient, their family, a colleague or junior member?
Have you witnessed a member of your team making disparaging or demeaning remarks about a patient, their family, a colleague or junior member behind their backs?
Have you witnessed low quality of patient care due to incompetence, poor team communication or lack of provider continuity?
Have you witnessed compromised patient care due to lack of resources, equipment, bed capacity or administrative support?
Have you witnessed stigmatizing social circumstances or conditions (e.g. alcoholism, drug abuse, homelessness, or obesity) leading to less than optimal care being provided?</td></tr>
<tr><td></td><td>Go to respective rings that were not covered in the case discussion above</td></tr>
<tr><td>Innate Ring</td><td>36. How do you view life and death?
For e.g., do you hold the view that life has inherent meaning?
For e.g., could you share with us what you think makes a “good life” or “good death”?
37. Have you always held this view? / Has this concept of life and death always been there or has it changed as you cared for dying patients?</td></tr>
</table>

	• For e.g., have you always been (against euthanasia)?<br><br>38. Are these concepts still evolving?<br>39. (Based on their personal conception of life / death)<br>• What are the principles or values that influence the way you think about life and death?<br>• Do you think these beliefs influence your decision-making and the actions that you take and if so, how?
**Relational Ring**	40. How do you view your relationships with people who matter to you?<br>• For e.g., your family, close friends, or your significant other?<br>• Some doctors / nurses we interviewed said that caring for dying patients makes them distance themselves for fear of loss.<br>41. Has this always been the case or has this changed in the course of caring for suffering/dying patients? (If it has changed, why?)
**Societal Ring**<br>*Professional Relationships*	42. How have these experiences affected how you view your role as a physician/APN?<br>43. How have these experiences changed the way you think about your roles and responsibilities as a physician / APN?<br>• For e.g., do you think you play God extubating patients, stopping feeding, etc.?<br>44. How does caring for suffering/dying patients affect the way you think about your **patients**?<br>45. How does caring for suffering/dying patients affect the way you think about your **patient's family**?<br>46. How does caring for suffering/dying patients affect the way you think about your colleagues?<br>47. How does caring for suffering/dying patients affect the way you think about people in **society at large**?<br>48. Do you think your views on euthanasia differs from your fellow colleagues?<br>• If yes, how do you think this affects your role as a physician?
**Individual Ring**	49. What are the skills you possess that you believe are important to have in caring for suffering/dying patients?<br>50. Have these been shaped by your experiences?<br>51. How do you view patients who are unable to express themselves, or who lack mental capacity or volition or those who are unconscious?<br>• For e.g., do you think they have rights or value? How do you respect them?<br>52. Has this always been the case or has this changed?

Additional File 2. Key Dimensions to Living Well

Key Point	Reported by	Supporting Quotes
Happiness and satisfaction	P5, P7, P11, P12, G1, G3, G7, G11, E2, E9	"I think the good life is seeking the pleasure of work, which of course involves this aspect of taking your share of this world, enjoying things that are enjoyable and pleasant in this world." (P11)
Good quality of life, including good health and environment	G2, G3, E12	"A good life is a life that is lived with good quality, namely able to function, able to mobilise without depending much on other people and to be in an environment which is comfortable and in a happy state of mind." (G3)
Independence and living life by one's own values, beliefs, experiences and relationships	P4-P10, P16, G1, G3, G4, G9, G10, G12, E3, E11	"I think good life [is…] very much depending on that person, what makes the person's values and what are the person's experiences in life. For example, for me, I find that I'm someone who does not like to burden my family." (P9)
Maintenance of dignity	P11, G10	"A good life is basically being content with whatever they have… And most importantly, having a very dignified life. (G10)"
Living closely to one's faith and spirituality and making appropriate preparation for the afterlife	P11, P16, G4, E7	"A good life is, I mean, as a Muslim, you have to follow what is in the religion… You must know why you are here… Why God created you… your purpose in life." (E7)
A meaningful life in the service of family and society, alongside making a difference to the community.	P5, P9, P10, P11, P15, G3, G4, G8, E3, E7, E9, E10	"Good life means, for me… surrounded by family members, maintain a good relationship with friends and family." (G4)
Achievement of personal goals	P8, P10, P15, G1,	"When we achieve what's expected to

and financial stability	G5, G9, E1, E6, E9, E13	be achieved by you." (P8)

Additional File 3. Key Dimensions to Dying Well

Key Point	Reported by	Supporting Quotes
Highly personalized and consistent with patient's perception and religious beliefs	P4, P5, P8-P10, P13, P16, G1, G2, E6	"A good death is when you die in a manner that you choose." (E6)
Patient's family is at peace and prepared for the patient's demise	P5, P6, G1, G7, G10, E2, E6, E9	"To make sure that when any older person is dying, they are kept most comfortable and not just them. It's also their family members, because often it's the family members who actually live with that guilt, seeing their loved ones, you know, suffering, gasping or in pain." (G10)
'Quick death' with dignity and/or free of physical, social, emotional, spiritual distress	P5, P6, P12, G1-G3, G5-G7, G10-G12, E1, E3, E7, E10, E11	"What makes a good death? I guess the least amount of suffering and a death that is dignified in its own way." (E3)
Resolved issues without regrets	P7, P12, G1, G11, E7	"What a good dying should be like… people should reconcile with things before they leave this world. And there will be an intentional effort to try to explore their regrets or unfinished business." (P7)
Establishment of their legacy	P6, E10	"If you could benefit the people around you, you could make your mark and leave a good impact to the world." (E10)
Death in the place of their choosing and in the presence of loved ones	P6, G1, G3, G5, G9, G10, G12, E12	"A good death will be an environment which is conducive for the person who is dying, with the people who care for him or her around that person." (G3)

Death as the only release from unbearable suffering	G2	"I feel it must be because of the immense amount of suffering, the physical or psychological that the person is experiencing and the person feels there is no way it can be relieved, except death." (G2)
